# Plasma Leptin Levels in Children Hospitalized with Cholera in Bangladesh

**DOI:** 10.4269/ajtmh.15-0172

**Published:** 2015-08-05

**Authors:** Brie Falkard, Taher Uddin, M. Arifur Rahman, Molly F. Franke, Amena Aktar, Muhammad Ikhtear Uddin, Taufiqur Rahman Bhuiyan, Daniel T. Leung, Richelle C. Charles, Regina C. Larocque, Jason B. Harris, Stephen B. Calderwood, Firdausi Qadri, Edward T. Ryan

**Affiliations:** Division of Infectious Diseases, Massachusetts General Hospital, Boston, Massachusetts; Center for Vaccine Sciences, International Centre for Diarrhoeal Disease Bangladesh (icddr,b), Dhaka, Bangladesh; Partners In Health, Boston, Massachusetts; Department of Global Health and Social Medicine, Harvard Medical School, Boston, Massachusetts; Department of Microbiology and Immunobiology, Harvard Medical School, Boston, Massachusetts; Department of Medicine, Harvard Medical School, Boston, Massachusetts; Division of Infectious Disease, University of Utah School of Medicine, Salt Lake City, Utah; Department of Pediatrics, Harvard Medical School, Boston, Massachusetts; Department of Immunology and Infectious Disease, Harvard School of Public Health, Boston, Massachusetts

## Abstract

*Vibrio cholerae*, the cause of cholera, induces both innate and adaptive immune responses in infected humans. Leptin is a hormone that plays a role in both metabolism and mediating immune responses. We characterized leptin levels in 11 children with cholera in Bangladesh, assessing leptin levels on days 2, 7, 30, and 180 following cholera. We found that patients at the acute stage of cholera had significantly lower plasma leptin levels than matched controls, and compared with levels in late convalescence. We then assessed immune responses to *V. cholerae* antigens in 74 children with cholera, correlating these responses to plasma leptin levels on day 2 of illness. In multivariate analysis, we found an association between day 2 leptin levels and development of later anti-cholera toxin B subunit (CtxB) responses. This finding appeared to be limited to children with better nutritional status. Interestingly, we found no association between leptin levels and antibody responses to *V. cholerae* lipopolysaccharide, a T cell–independent antigen. Our results suggest that leptin levels may be associated with cholera, including the development of immune responses to T cell–dependent antigens.

## Introduction

*Vibrio cholerae* is a Gram negative organism and the cause of cholera, a severe dehydrating illness of humans.^1^ It is estimated that approximately 3–5 million individuals develop cholera each year and that approximately 100,000 people die of cholera annually.^2^ The burden of cholera is highest in areas of the world with severe poverty and lack of access to safe water and adequate nutrition. Many of the individuals who are at risk for cholera are also afflicted by other health conditions, including nutritional deficiencies.

Leptin is a hormone that is involved in both metabolism and mediating immune responses during human infection.^3^ Leptin is primarily released into the plasma by adipose tissue, but is also produced by gastric and colonic epithelial cells and T cells during acute inflammation.^4^ Undernourished individuals have lower leptin levels in the circulation than well-nourished individuals.^5^ In general, males have lower leptin levels than females, perhaps reflecting differences in the amount and distribution of adipose tissue.^6^ The receptor for the leptin molecule is expressed on a number of different cell types, including intestinal epithelial and immune cells, such as macrophages, T cells, natural killer cells, and polymorphonuclear leukocytes, as well as other cell types such as neurons.^7^ Alterations in the leptin receptor and leptin gene expression have been associated with changes in immune responses and increased susceptibility to infection.^8^ Since cholera often occurs in populations with undernourishment or other nutritional deficiencies, we were interested in characterizing plasma leptin levels in children with cholera who were 5 years of age or younger in Dhaka, Bangladesh, and the association of these levels with subsequent immune responses. We hypothesized that leptin levels would increase in response to cholera infections, acting as an acute inflammatory cytokine.

## Methods

### Study subjects.

In this study, we enrolled 74 participants (6–60 months of age) who were admitted to the Dhaka Hospital of the International Center for Diarrheal Disease Research, Bangladesh (ICDDR,B) after presenting with severe acute watery diarrhea and whose stool was positive for *V. cholerae* O1 by microbiologic culture.^9^ After clinical stabilization, we collected blood samples by venipuncture from study participants on day 2, as well as anthropometric measurements. Blood samples were used for ABO typing and baseline vibriocidal measurement on enrollment in the study. We collected additional blood samples on days 7 and 30. We also collected day 180 samples from a subset (*N* = 11) of these patients who were enrolled in a separate study that collected day 180 venipuncture samples. To match these 11 cases, we enrolled 11 controls who were matched for gender, age (±6 months), and nutritional weight-for-age (WAZ) status, collecting a single baseline blood sample from these control participants.

Classification of nutritional status via Z score was based on World Health Organization (WHO) anthropometric classifications (http://www.who.int/childgrowth/software/en/). We categorized children by Z score for the following nutritional categories: weight-for-height (WAZ), weight-for-height (WHZ), and height-for-age (HAZ). We classified children according to the WHO definitions for undernutrition. We classified children with a WAZ score greater or equal to −2 as not moderately/severely undernourished, children with a WAZ score between −2 and −3 as moderately undernourished, and children with a WAZ score lower than −3 as severely undernourished. We classified children with a WHZ score greater or equal to −2 as not moderately/severely wasted, children with a WHZ score between −2 and −3 as moderately wasted, and children with a WHZ score lower than −3 as severely wasted. We classified children with a HAZ score greater or equal to −2 as not moderately/severely stunted, children with a HAZ score between −2 and −3 as moderately stunted, children with an HAZ score lower than −3 as severely stunted. This study was approved by the institutional review boards of the ICDDR,B and Massachusetts General Hospital, Boston, MA.

### Leptin assessment in sample plasma.

We determined the concentration of leptin in the plasma of patients and healthy controls using a commercially available Human Leptin enzyme-linked immunosorbent assay (ELISA) Kit, as per the manufacturer’s instructions (Cat. No. RAB0333; Sigma Aldrich, St. Louis, MO).

### Antibody detection in patient plasma.

We assessed plasma samples for cholera toxin-B subunit (CtxB) and lipopolysaccharide (LPS)–specific antibodies of the IgG and IgA isotypes using standardized ELISA as previously described.^10^,^11^ Specifically, we used plates coated with ganglioside GM1 (Sigma G9652) and CtxB (Sigma C9903) or *V. cholerae* O1 LPS (2.5 μg/mL). We detected human antibody binding using mouse biotinylated monoclonal antibodies (1:2,000 dilution; Jackson ImmunoResearch, West Grove, PA) and streptavidin-conjugated horseradish peroxidase (1:4,000 dilution; Amersham, Piscataway, NJ). We developed plates with 3,3′,5,5′-tetramethylbenzidine (Sigma) and 0.01% H_2_SO_4_ (50 μL/well), and assessed absorbance at 450 nm in a kinetic ELISA system.^12^

### Statistical analysis.

We used paired *t* tests to assess differences in plasma leptin levels in 11 cholera patients across time points (days 2, 7, 30, and 180) and between these cholera patients and 11 matched controls. In 74 children with cholera, we examined whether day 2 leptin values were associated with subsequent antibody responses using linear regression, with CtxB antibody values transformed to the square-root scale to achieve a normal distribution. To account for factors that may influence both leptin and CtxB antibody values (i.e., potential confounders), we included the following covariates in a multivariate regression model: vibriocidal titer, blood group, age, gender, and nutritional status (WAZ, WHZ, and HAZ score). We assessed collinearity between the nutritional categories by examination of the variance inflation factor. Analysis of anti-LPS responses was similarly performed. Finally, to examine whether any observed association between leptin and CtxB and LPS antibody values depended on nutritional status or gender, we examined these correlations graphically across categories of WAZ, WHZ, and HAZ and gender. Analyses were conducted using Stata (Version 13.1; StataCorp, College Station, TX) and GraphPad software (Version 5.0d; La Jolla, CA), and we considered *P* values < 0.05 as statistically significant.

## Results

### Leptin levels during cholera.

**Figure 1.**
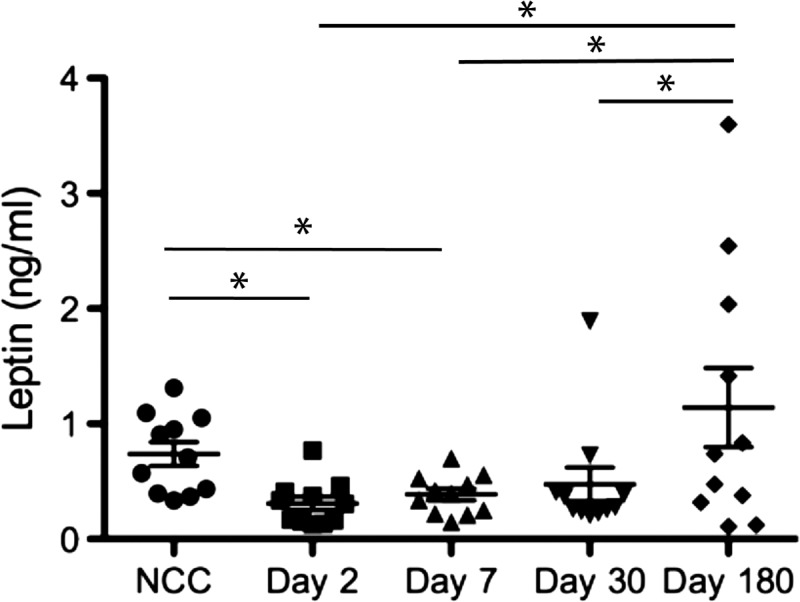
Mean plasma leptin levels on days 2, 7, 30, and 180 in children with cholera in Bangladesh and non-cholera controls (NCCs) matched for age, gender, and weight-for-age (WAZ). * *P* < 0.05. Error bars represent standard error of the mean.

We found that leptin levels in plasma from 11 patients were significantly reduced during the acute phase of cholera infection compared with subsequent days of infection, contrary to our initial hypothesis (Figure 1

). Specifically, leptin levels collected on days 2, 7, and 30 were significantly lower than day 180 levels (*P* < 0.05). When we compared plasma leptin levels in these 11 cholera patients to age, gender, and nutritionally (WAZ) matched controls (Table 1), we found that the plasma leptin levels during the acute phase of cholera (days 2 and 7) were significantly lower than the levels in non-cholera matched controls (*P* < 0.05) (Figure 1). We also found the same trend of reduced leptin levels in all 74 participants (47% of the participants were female, 53% were male) within the acute phase of cholera (days 2 and 7) in comparison to day 30 and healthy controls.

### Association of leptin levels with immune responses to cholera.

**Figure 2.**
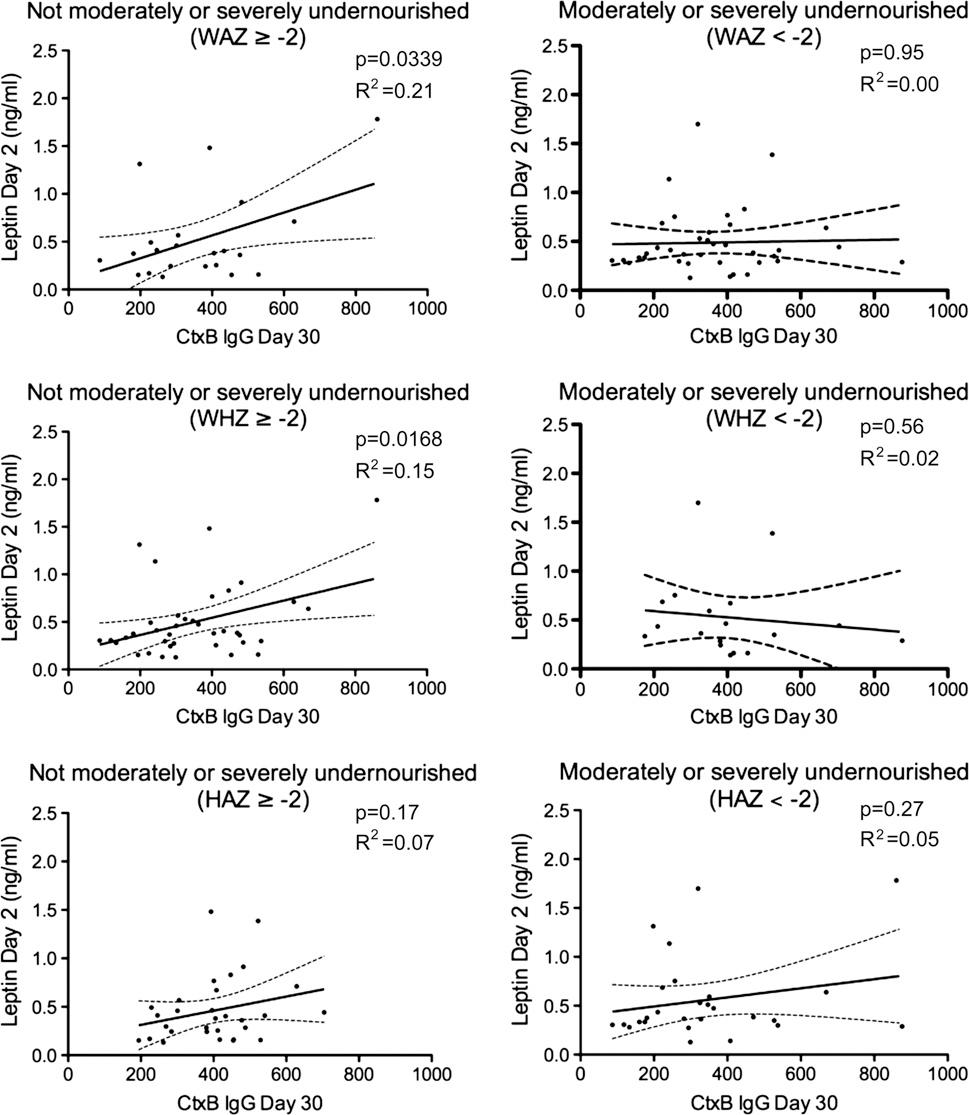
Correlation between day 2 plasma leptin and day 30 IgG responses to the cholera toxin-B subunit (CtxB). Nutritional categorization on day 2 of cholera patients. WAZ = weight-for-age; WHZ = weight-for-height; HAZ = height-for-age, as described by World Health Organization anthropometric classifications (http://www.who.int/childgrowth/software/en/). Children with a Z score < −2 were categorized as moderately or severely undernourished for that category. Children with a Z score ≥ −2 were categorized as non-moderately or severely undernourished. *P* value and *R*^2^ values are shown.

**Figure 3.**
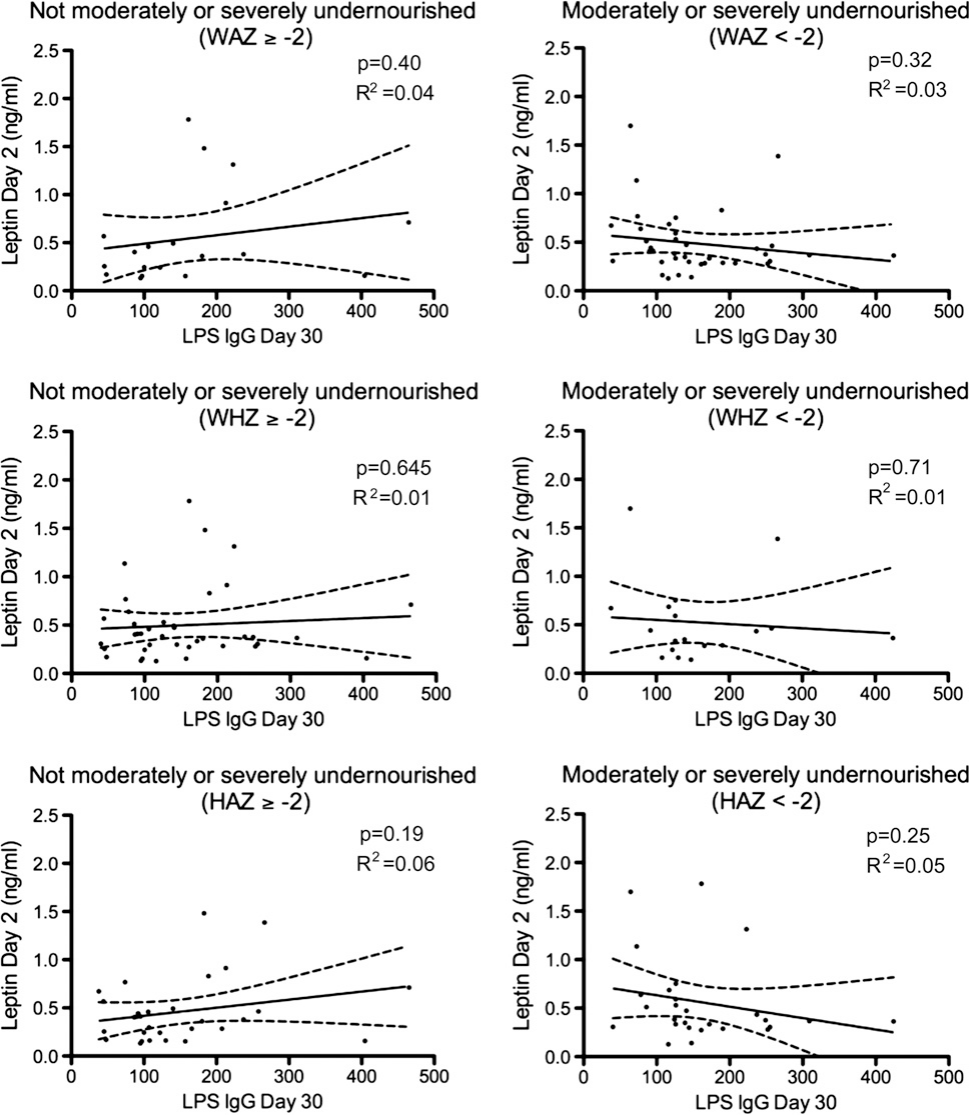
Correlation between leptin on days 2 and 30 IgG responses to *Vibrio cholerae* O1 lipopolysaccharide (LPS). Nutritional categorization on day 2 of cholera patients. WAZ = weight-for-age; WHZ = weight-for-height; HAZ = height-for-age, as described by World Health Organization anthropometric classifications (http://www.who.int/childgrowth/software/en/). Children with a Z score < −2 were categorized as moderately or severely undernourished for that category. Children with a Z score ≥ −2 were categorized as non-moderately or severely undernourished. *P* value and *R*^2^ values are shown.

In a multivariate analysis incorporating day 2 leptin level, gender, age, vibriocidal titer, nutritional status (WAZ, WHZ, and HAZ) and blood group, we found a statistically significant positive association between normalized (square-root adjusted) day 30 CtxB antibody responses and day 2 plasma leptin levels (*P* = 0.05) and HAZ status (*P* = 0.02) (Table 2). We found no such association when we reran the model assessing associations with day 30 plasma LPS antibody levels (Supplemental Table 1). When we stratified children by nutritional status, we found that day 2 plasma leptin levels correlated with subsequent development of IgG antibody responses to CtxB on day 30 only among children who were not moderately or severely undernourished by WAZ and WHZ (Figure 2

). We did not observe this correlation in subgroups of HAZ scores or in moderately or severely undernourished children according to WAZ or WHZ (Figure 2). As in the multivariate analyses, we did not find any association between day 2 plasma leptin levels and subsequent development of IgG antibody responses targeting LPS among subgroups characterized by nutritional status (Figure 3

). We found a trend toward an association between day 2 leptin levels and subsequent IgA responses to CtxB in not moderately or severely undernourished children when considering WHZ score (*P* = 0.076; data not shown), but no association with LPS responses in any cohort. In females who were not moderately or severely undernourished by WAZ or WHZ, we similarly found an association of day 2 leptin levels and subsequent development of IgA responses to CtxB, but not to LPS (Supplemental Figures 1 and 2). This association was not found in male participants.

## Discussion

To our knowledge, this study is the first assessment of leptin levels in individuals with cholera. We found that leptin levels are low during the acute stage of cholera compared with levels in healthy matched controls and that plasma leptin levels rise following cholera during late convalescence. Our observations may not have been predicted based on previous data. Previous work has suggested that leptin can be considered an acute-phase reactant and may be elevated during acute disease. For instance, in mice, plasma leptin levels increase acutely after the administration of pro-inflammatory cytokines (such as interleukin 1 [IL-1] and tumor necrosis factor alpha [TNF-α]) and in response to infectious stimuli such as LPS.^5^ Other studies have demonstrated an increase in leptin expression in infected tissues, such as in the lung of mice infected with *Mycobacterium tuberculosis*.^13^ Although *V. cholerae* is a noninvasive luminal pathogen, recent data suggest that intestinal infection with *V. cholerae* is also associated with a pro-inflammatory response.^14^,^15^ Whether our results suggest that plasma leptin levels fall during the acute stage of cholera, or that a low plasma leptin level places a child at risk of developing clinically symptomatic disease on exposure to *V. cholerae*, is not clear at present. Similarly, since anthropometric measurements were not performed at day 180 in this study, we cannot answer whether the rise in plasma leptin levels in cholera patients by late convalescence reflected recovery from cholera or improvement in nutritional status of affected children. Another possibility is that exposure to basic medical care could have impacted the subsequent household care provided to the participants and improved their nutritional status following their illness.

Our data also suggest that leptin plays a role in the development of antibody responses to cholera antigens. In this study, we found a significant association between day 2 plasma leptin levels and convalescent-phase plasma IgG antibody responses to CtxB, a T cell–dependent antigen. We did not find any association between leptin and IgG responses to LPS, a T cell–independent antigen. Previous studies have demonstrated deficiencies in T cell–mediated responses in undernourished children with low plasma leptin levels, including a significantly lower number of peripherally circulating CD4^+^ T cells and a lower T helper cells Th1:Th2 ratio.^16^ An increase in the plasma leptin levels with weight gain has also been associated with both an increase in the number of peripherally circulating CD4^+^ T and an increase in their Th1 profile.^16^,^17^ The long form of the leptin receptor, LepRb, is expressed on T cells, where it signals through pathways found in multiple cell types. For instance, binding by leptin leads to JAK2 phosphorylation of LepRb and activation of several transcription factors, including STAT3, STAT5, STAT6, and SHP-2 and the ERK-MAPK signaling pathway,^18^ and these pathways promote lineage commitment of CD4 + cells and production of interferon gamma (IFN-γ) by Th1 cells.^19^ Leptin can also directly regulate cytokine production in lymphocytes; administration of exogenous leptin to T cells in vitro increases IFN-γ and IL-2 production while decreasing IL-4 levels.^20^ Alterations to IFN-γ production regulate Th1/Th2 balance and impact B-cell activation since IFN-γ secretion from Th cells can be a critical cytokine during B-cell activation in germinal centers.^18^,^21^ Transcription factors SHP-2 and STAT-5 also play a role in mammalian cell anti-apoptosis pathways,^22^ and leptin’s activation of this anti-apoptotic pathway has been shown to mediate resistance to the invasive enteric pathogen, *Entamoeba histolytica*.^7^ A human genome study for single nucleotide polymorphisms (SNPs) associated with resistance to *Entamoeba histolytica* also identified a Q223R mutation in the *LepR* gene,^8^ and mice with the mutant receptor have been shown to be more susceptible to *Entamoeba histolytica* infection and have greater levels of apoptosis of intestinal epithelial cells (IECs).^8^,^23^ Although *V. cholerae* is a noninvasive pathogen, it does secrete cholera toxin (CT), a potent enterotoxin, and CT has also been associated with apoptosis in certain cell types.^24^ Our observed association of plasma leptin levels with antibody responses to CtxB but not to LPS may thus further support a role of leptin in mediating T-cell immunity during cholera.

In our study, we detected an association of day 2 leptin levels with anti-CtxB antibody levels among children who were not undernourished by WAZ and WHZ category, but not by HAZ. These findings may be a reflection of our relatively small cohort size when stratified by nutritional status, but it is also possible that WAZ and WHZ may be more sensitive markers of recent undernutrition. The higher correlation of plasma leptin levels to body fat percentage in non-malnourished or after weight gain in undernourished children, may explain in part why we found a correlation in day 2 leptin levels and subsequent antibody responses only in not undernourished children.^25^

In our multivariate analysis, stunting (HAZ score < −2) was independently and positively associated with CtxB responses in addition to the association of the acute-stage leptin level and CtxB responses. This was a surprising result since we did not find an association between leptin levels and CtxB responses when stratifying children by HAZ score. Once again, this may reflect our relatively small cohort size when stratified by nutritional status; however, stunting in the children in this study may in part reflect the sequelae of environmental (tropical) enteropathy^26^,^27^ resulting from frequent and recurrent intestinal infections, including by CT-expressing *V. cholerae* and immunologically related heat labile toxin (LT)–expressing enterotoxigenic *Escherichia coli* (ETEC), and therefore possibly may reflect previous exposure and immunological priming to CT/LT in these children.

There are several significant limitations to our study. First, our study is descriptive and not mechanistic. We also did not assess leptin levels in children prior to their becoming symptomatic with cholera, so we cannot comment on whether leptin levels affect the risk of cholera or change significantly with onset of acute disease. We also only assessed leptin levels in plasma and not in intestinal tissue during cholera, and our sample size is also relatively small, especially when considering children by various nutritional categories. Despite these limitations, our study of 74 children with cholera in Bangladesh suggests an association of plasma leptin levels and cholera and an association of leptin with immune responses during cholera. We feel that these findings are significant. Future work to expand our observations could include assessing leptin levels during other bacterial intestinal infections, assessing possible association with leptin and antibody maturation and class switching, assessing a possible role of leptin to T cell–dependent and T cell–independent antigens beyond those used in this study, assessing whether leptin levels predispose to cholera itself or to severity of disease, assessing leptin levels in mucosal tissues during intestinal infection, and addressing potential mechanisms of leptin involvement in immune responses during cholera. Importantly, our data not only suggest that leptin levels may be associated with cholera, including the level of immune responses to T cell–dependent antigens, but also suggest that leptin levels may remain suppressed for at least a month after clinical recovery from cholera, raising the possibility that the nutritional and immunologic impact of an episode of cholera may extend long after the period of apparent clinical stabilization, rehydration, and resolution of diarrhea.

## Supplementary Material

Supplemental Datas.

## Figures and Tables

**Table 1:** Characteristics of the 11 study participants with matched NCCs

	Cholera-infected children (*N* = 11)	Matched non-cholera controls (*N* = 11)	*P* value
Mean age in months ± SD	44.7 ± 14	47.6 ± 7.5	0.83
Number of females (%)	6 (55)	6 (55)	1.00
WAZ ≥ −2	3 (27)	3 (27)	1.00
WHZ ≥ −2	7 (64)	7 (64)	1.00
HAZ ≥ −2	8 (73)	3 (27)	0.416

HAZ = height-for-age; NCCs = non-cholera controls; SD= standard deviation; WAZ = weight-for-age, as described by World Health Organization anthropometric classifications (http://www.who.int/childgrowth/software/en/); WHZ = weight-for-height.

Nutritional categorization on day 2 of cholera patients and day 0 of NCCs.

**Table 2:** Bivariate and multivariate models: linear regression of square-root CtxB IgG day 30 antibody titer and additional variables of 74 participants

	Bivariate model			Multivariable model		
	Coef.	95% CI	*P* value	Coef.	95% CI	*P* value
Leptin day 2	1.54	−1.05, 4.13	0.18	2.9	−0.09, 6.08	0.05
WAZ nutritional category	0.63	−1.67, 2.92	0.58	−0.04	−3.05, 2.96	0.98
WHZ nutritional category	−1.41	−3.70, 0.89	0.23	−0.64	−3.23, 1.94	0.62
HAZ nutritional category	1.58	−0.56, 3.73	0.15	3.43	0.64, 6.23	0.02
Gender	−0.36	−2.54, 1.82	0.74	−2.20	−4.55, 0.15	0.06
Age	0.01	−0.08, 0.09	0.89	0.03	−0.06, 0.13	0.49
Blood group	1.63	−0.5, 3.78	0.13	1.99	−0.20, 4.20	0.07
Vibriocidal day 2	0.01	−0.002, 0.005	0.35	0.00	−0.001, 0.005	0.37

Coef = coefficient variable; CI = confidence interval; CtxB = cholera toxin-B subunit; HAZ = height-for-age; WAZ = weight-for-age; WHZ = weight-for-height.

## References

[1] Harris JB, LaRocque RC, Qadri F, Ryan ET, Calderwood SB (2012). Cholera. Lancet.

[2] WHO (2010). WHO position paper on cholera vaccines. Wkly Epidemiol Rec.

[3] Procaccini C, Pucino V, De Rosa V, Marone G, Matarese G (2014). Neuro-endocrine networks controlling immune system in health and disease. Front Immunol.

[4] Sobhani I, Bado A, Vissuzaine C, Buyse M, Kermorgant S, Laigneau J-P, Attoub S, Lehy T, Henin D, Mignon M, Lewin MJ (2000). Leptin secretion and leptin receptor in the human stomach. Gut.

[5] Faggioni R, Feingold KR, Grunfeld C (2001). Leptin regulation of the immune response and the immunodeficiency of malnutrition. FASEB J.

[6] Garcia-Mayor RV, Andrade MA, Rios M, Lage M, Dieguez C, Casanueva FF (1997). Serum leptin levels in normal children: relationship to age, gender, body mass index, pituitary-gonadal hormones, and pubertal stage. J Clin Endocrinol Metab.

[7] Mackey-Lawrence NM, Petri WA (2012). Leptin and mucosal immunity. Mucosal Immunol.

[8] Duggal P, Guo X, Haque R, Peterson KM, Ricklefs S, Mondal D, Alam F, Noor Z, Verkerke HP, Marie C, Leduc CA, Chua SC, Myers MG, Leibel RL, Houpt E, Gilchrist CA, Sher A, Porcella SF, Petri WA (2011). A mutation in the leptin receptor is associated with *Entamoeba histolytica* infection in children. J Clin Invest.

[9] Weil AA, Chowdhury F, Khan AI, Leung DT, Uddin T, Begum YA, Saha NC, Charles RC, LaRocque RC, Harris JB, Ryan ET, Qadri F, Calderwood SB (2012). Frequency of reexposure to *Vibrio cholerae* O1 evaluated by subsequent vibriocidal titer rise after an episode of severe cholera in a highly endemic area in Bangladesh. Am J Trop Med Hyg.

[10] Qadri F, Wenneras C, Albert MJ, Hossain J, Mannoor K, Begum YA, Mohi G, Salam MA, Sack RB, Svennerholm AM (1997). Comparison of immune responses in patients infected with *Vibrio cholerae* O139 and O1. Infect Immun.

[11] Qadri F, Ahmed F, Karim MM, Wenneras C, Begum YA, Salam MA, Albert MJ, McGhee JR (1999). Lipopolysaccharide- and cholera toxin-specific subclass distribution of B-cell responses in cholera. Clin Diagn Lab Immunol.

[12] Rahman A, Rashu R, Bhuiyan TR, Chowdhury F, Khan AI, Islam K, LaRocque RC, Ryan ET, Wrammert J, Calderwood SB, Qadri F, Harris JB (2013). Antibody-secreting cell responses after *Vibrio cholerae* O1 infection and oral cholera vaccination in adults in Bangladesh. Clin Vaccine Immunol.

[13] Wieland CW, Florquin S, Chan ED, Leemans JC, Weijer S, Verbon A, Fantuzzi G, van der Poll T (2005). Pulmonary *Mycobacterium tuberculosis* infection in leptin-deficient ob/ob mice. Int Immunol.

[14] Uddin T, Harris JB, Bhuiyan TR, Shirin T, Uddin MI, Khan AI, Chowdhury F, LaRocque RC, Alam NH, Ryan ET, Calderwood SB, Qadri F (2011). Mucosal immunologic responses in cholera patients in Bangladesh. Clin Vaccine Immunol.

[15] Arifuzzaman M, Rashu R, Leung DT, Hosen MI, Bhuiyan TR, Bhuiyan MS, Rahman MA, Khanam F, Saha A, Charles RC, LaRocque RC, Weil AA, Clements JD, Holmes RK, Calderwood SB, Harris JB, Ryan ET, Qadri F (2012). Antigen-specific memory T cell responses after vaccination with an oral killed cholera vaccine in Bangladeshi children and comparison to responses in patients with naturally acquired cholera. Clin Vaccine Immunol.

[16] Palacio A, Lopez M, Perez-Bravo F, Monkeberg F, Schlesinger L (2002). Leptin levels are associated with immune response in malnourished infants. J Clin Endocrinol Metab.

[17] Rodriguez L, Graniel J, Ortiz R (2007). Effect of leptin on activation and cytokine synthesis in peripheral blood lymphocytes of malnourished infected children. Clin Exp Immunol.

[18] Frühbeck G (2006). Intracellular signalling pathways activated by leptin. Biochem J.

[19] Rincón M (2001). MAP-kinase signaling pathways in T cells. Curr Opin Immunol.

[20] Fantuzzi G, Sennello JA, Batra A, Fedke I, Lehr HA, Zeitz M, Siegmund B (2005). Defining the role of T cell-derived leptin in the modulation of hepatic or intestinal inflammation in mice. Clin Exp Immunol.

[21] Cerutti A, Puga I, Cols M (2012). New helping friends for B cells. Eur J Immunol.

[22] Marie CS, Verkerke HP, Paul SN, Mackey AJ, Petri WA (2012). Leptin protects host cells from *Entamoeba histolytica* cytotoxicity by a STAT3-dependent mechanism. Infect Immun.

[23] Guo X, Roberts MR, Becker SM, Podd B, Zhang Y, Chua SC, Myers MG, Duggal P, Houpt ER, Petri WA (2010). Leptin signaling in intestinal epithelium mediates resistance to enteric infection by *Entamoeba histolytica*. Mucosal Immunol.

[24] Allam M, Bertrand R, Zhang-Sun G, Pappas J, Viallet J (1997). Cholera toxin triggers apoptosis in human lung cancer cell lines. Cancer Res.

[25] Palacio A, Lopez M, Perez-Bravo F, Monkeberg F, Schlesinger L (2002). Leptin levels are associated with immune response in malnourished infants. J Clin Endocrinol Metab.

[26] Keusch GT, Rosenberg IH, Denno DM, Duggan C, Guerrant RL, Lavery JV, Tarr PI, Ward HD, Black RE, Nataro JP, Ryan ET, Bhutta ZA, Coovadia H, Lima A, Ramakrishna B, Zaidi AKM, Burgess DCH, Brewer T (2013). Implications of acquired environmental enteric dysfunction for growth and stunting in infants and children living in low- and middle-income countries. Food Nutr Bull.

[27] Keusch GT, Denno DM, Black RE, Duggan C, Guerrant RL, Lavery JV, Nataro JP, Rosenberg IH, Ryan ET, Tarr PI, Ward H, Bhutta ZA, Coovadia H, Lima A, Ramakrishna B, Zaidi AKM, Hay Burgess DC, Brewer T (2014). Environmental enteric dysfunction: pathogenesis, diagnosis, and clinical consequences. Clin Infect Dis.

