# Supplementary material for: Plasma Leptin Levels in Children Hospitalized with Cholera in Bangladesh
Source: Am J Trop Med Hyg. 2015 Aug 5;93(2):244–9. doi: 10.4269/ajtmh.15-0172 (PMC4530742; doi:10.4269/ajtmh.15-0172)
Supplement: Supplementary file 1 [file SD3.pdf]

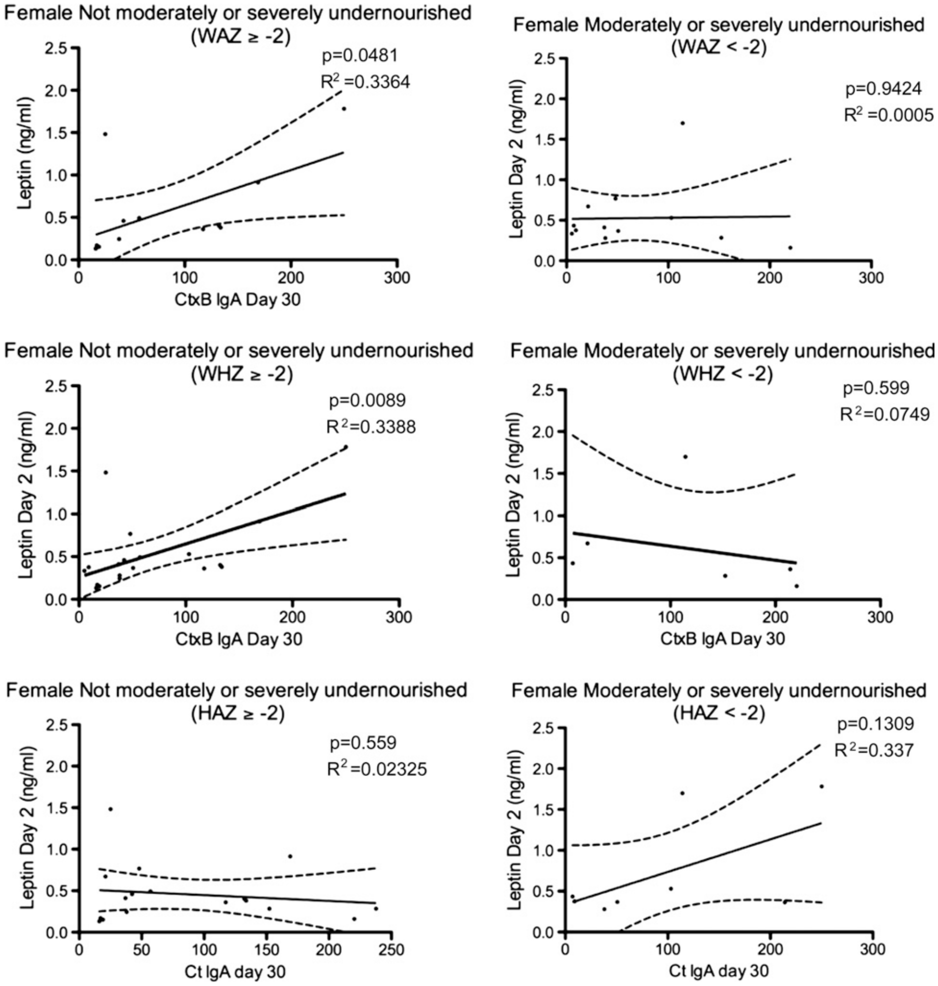

SUPPLEMENTAL FIGURE 1. Stratification by nutritional classifications of female cholera patients, correlating plasma leptin levels on day 2 vs. IgA antibody responses to cholera toxin-B subunit (CtxB) on day 30. Nutritional categorization on day 2 of cholera patients: WAZ = weight-for-age; WHZ = weight-for-height; HAZ = height-for-age, as described by World Health Organization anthropometric classifications (http://www.who.int/childgrowth/software/en/). Children with a Z score < −2 were categorized as moderately or severely undernourished for that category. Children with a Z score ≥ −2 were categorized as non-moderately or severely undernourished. *P* value and R^2 values are shown.

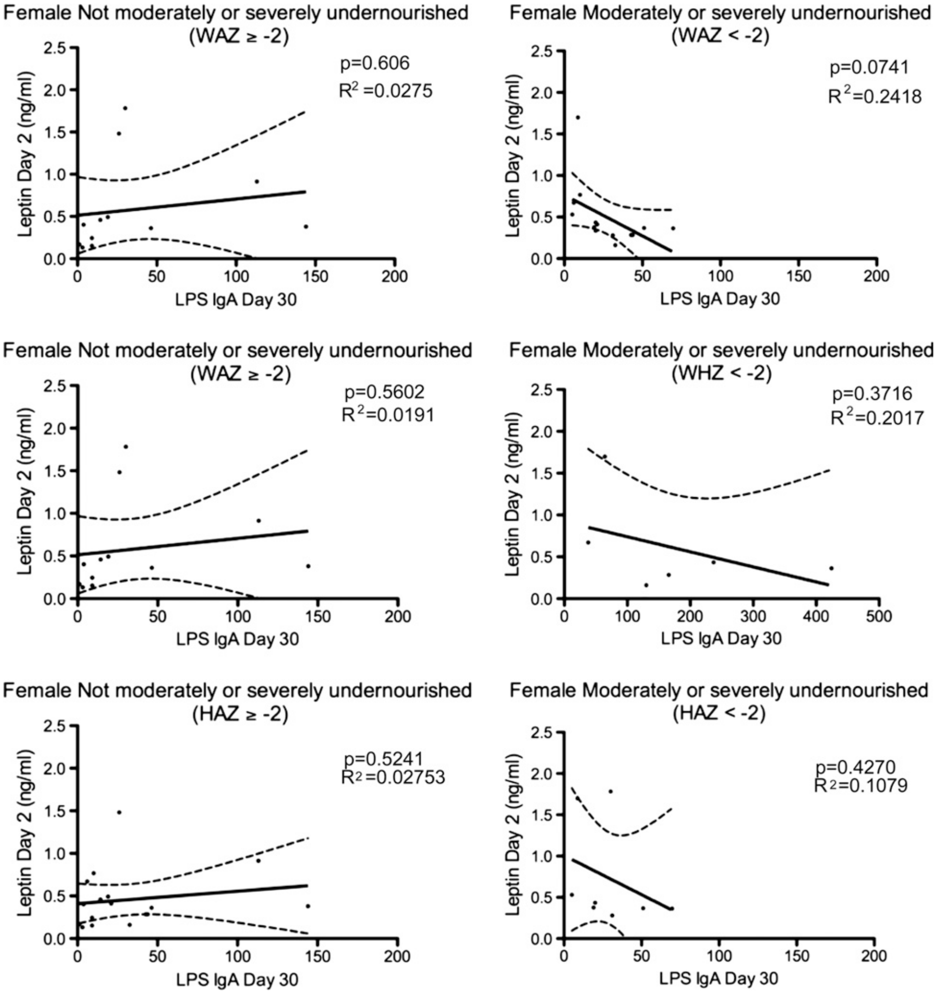

Supplemental Figure 2. Stratification by nutritional classifications of female cholera patients, correlating plasma leptin levels on day 2 vs. IgA antibody responses to *Vibrio cholerae* lipopolysaccharide (LPS) on day 30. Nutritional categorization on day 2 of cholera patients: WAZ = weight-for-age; WHZ = weight-for-height; HAZ = height-for-age, as described by World Health Organization anthropometric classifications (http://www.who.int/childgrowth/software/en/). Children with a Z score < −2 were categorized as moderately or severely undernourished for that category. Children with a Z score ≥ −2 were categorized as non-moderately or severely undernourished. *P* value and R^2 values are shown.

Supplemental Table 1

Bivariate and multivariate models: linear regression of square-root LPS IgG day 30 antibody titer and additional variables of 74 study participants

	Bivariate model			Multivariable model		
	Coef.	95% CI	*P* value	Coef.	95% CI	*P* value
Leptin day 2	1.10	−1.07, 3.28	0.31	0.4	−2.26, 3.21	*
WAZ nutritional category	0.012	−2.02, 2.04	0.99	0.05	−2.62, 2.71	–
WHZ nutritional category	−0.78	−2.83, 1.26	0.45	0.35	−1.94, 2.64	–
HAZ nutritional category	−0.81	−2.73, 1.10	0.15	−0.28	−2.76, 2.19	–
Gender	0.35	−1.58, 2.27	0.72	−0.03	−2.12, 2.05	–
Age	0.03	−0.05, 0.10	0.49	0.01	−0.08, 0.10	–
Blood group	−0.93	−2.84, 0.98	0.33	−0.17	−2.12, 1.78	–
Vibriocidal day 2	0.01	−0.002, 0.004	0.54	0.00	−0.003, 0.003	–

Coef = coefficient variable; CI = confidence interval; HAZ = height-for-age; LPS = lipopolysaccharide; WAZ = weight-for-age; WHZ = weight-for-height.
* All values are not significant.
